# Current Trends in the Duration of Anticoagulant Therapy for Venous Thromboembolism: A Systematic Review

**DOI:** 10.7759/cureus.18992

**Published:** 2021-10-23

**Authors:** Peter Alexander, Shakthi Visagan, Reem Issa, Vasavi Rakesh Gorantla, Sneha E Thomas

**Affiliations:** 1 Anatomical Sciences, St. George's University School of Medicine, St. George's, GRD; 2 Internal Medicine, University of Maryland Medical Center, Baltimore, USA

**Keywords:** deep vein thrombosis, pulmonary embolism, recurrent thrombosis, anticoagulation therapy, venous thromboembolism

## Abstract

Anticoagulation therapy is the first line and drug of choice for both the treatment and prophylaxis of venous thromboembolism (deep vein thrombosis and/or pulmonary embolism). Anticoagulation drugs, ranging from different preparations of heparin, warfarin, and newer direct oral drugs such as rivaroxaban and dabigatran, work mainly by inhibiting important factors and enzymes in the coagulation cascade by preventing fibrin formation, platelet aggregation, and clot assembly. With recurrent thrombosis and embolisms being a feared complication for many physicians treating such cases, anticoagulation is often extended beyond the initial three- to six-month acute phase after an incident of venous thromboembolism. For some groups of patients, anticoagulation needs to be offered indefinitely to decrease the risk of a recurrent thrombosis. However, this concomitantly increases obvious and dangerous adverse effects such as increased risk of hemorrhage, as the ability to clot is hindered. This tradeoff between recurrent venous thromboembolism and bleeding is what underscores the controversy of the clinical question: for how long should anticoagulation be administered for venous thromboembolism? This review analyzes the use of anticoagulants in different types of venous thromboembolism and remarks on current consensus and trends on the length of anticoagulation treatment. We are doing so while acknowledging that venous thromboembolism management is an active area of research that is rapidly evolving. A literature search was performed looking at recent studies on anticoagulant administration for the treatment of venous thromboembolism with a focus on varying durations and patient populations. Factors that affect clinical decisions of duration are also elucidated. The most clinically relevant anticoagulants were discussed and their effects on the risk of recurrent thrombosis and embolism, and the risk of bleeding in relation to other drugs were analyzed. Ultimately, this article discussed patterns of anticoagulant treatments duration and which patient groups are likely to benefit the most from certain durations, shedding light on the ambiguity in how physicians should approach administering anticoagulation therapy over time for a broad range of presentations of venous thromboembolism.

## Introduction and background

Post coronary artery disease (CAD) or myocardial infarction (MI) and stroke, venous thromboembolism (VTE) is the third most common cardiovascular condition. It affects approximately 1-2% of individuals in the U.S. annually, presenting with not only a high mortality, but also a high morbidity and recurrence rate. VTE, an umbrella term that categorizes the diseases deep vein thrombosis (DVT) and pulmonary embolism (PE) or their concomitant occurrence in patients, is a condition where blood abnormally clots in the deep veins of the human body, usually in the veins of the legs, and the subsequent thrombus ends up embolizing the pulmonary circulation. VTEs can be categorized in several ways: proximal DVTs (PDVTs) occur when thromboses originate from the deep veins of the legs above the knee, such as the femoral, popliteal, or the iliac veins. Isolated distal DVTs (IDDVTs) occur when the thromboses originate in the calf veins below the knee. VTEs are also categorized based on their relationship with certain risk factors; if a VTE can be traced to a known attributable cause such as an environmental risk factor (long distance travel, pregnancy, trauma or fracture, cancer, etc.) or an iatrogenic cause (immobilization in the hospital, catheterization, surgery, etc.) it is deemed as a provoked VTE and unprovoked otherwise [1]. VTEs are also categorized based on the reversibility or the persistence of those risk factors. A risk factor for VTE is referred to as transient if the risk is reversible, such as VTEs due to surgeries or acute illnesses. Persistent risk factors, on the other hand, include those that are not acutely reversible, such as an active and malignant cancer or obesity. Finally, transient risk factors are further grouped based on grade: major transient risk factors include surgeries that are longer than 30 minutes or pregnancy and minor risk factors include surgeries shorter than 30 minutes or prolonged immobilization experienced on a long flight. Because the underlying pathology of the disease is abnormal coagulation, whether due to venous stasis or vascular endothelial injury, the mainstay of treatment is anticoagulation therapy.

Traditionally, initial treatment for acute VTE has been intravenous (IV) administration of unfractionated heparin (UFH) for five to seven days; after a week, vitamin K antagonists (VKAs) are administered for a total of 10 to 14 days of combined initial anticoagulation. Length of treatment with anticoagulation after this acute period has been a controversial topic traditionally and depends on many of these same categorizations including provoked vs unprovoked, initial VTE vs recurrence, and risk factors. VKA therapy is usually continued for three months and there is evidence that the stoppage of anticoagulants before this time significantly increases the risk of recurrent VTE [2-4]. Currently, non-vitamin K-antagonist-oral-anticoagulants or novel-oral-anticoagulants (NOACs) (e.g. dabigatran, rivaroxaban, apixaban, and edoxaban), sometimes called direct oral anticoagulants (DOACs), in the initial and long-term treatment in non-oncological cases are recommended over VKA therapy [5]. NOACs appear to have similar efficacy in reducing recurrence risk of VTE, reduce bleeding risk, and are more convenient for patients when compared to VKA therapy [6]. After the acute phase of thrombosis has resolved, the patients are reassessed and placed on one of three possible management tracts; cessation of anticoagulant treatment, long-term therapy (six or 12 months, up to a maximum of two years), or indefinite therapy. Usually, in provoked VTE cases, anticoagulation is recommended for at least three months or until the provoking factor, such as continued bed rest, is no longer presenting any risk for a recurrent VTE; in unprovoked VTEs, anticoagulation is often provided indefinitely because, despite the increase in the risk of bleeding, the risk of recurrent VTE markedly decreases [7]. In general, some physicians find it difficult to stop anticoagulation treatment early or at three months, even in provoked cases where the risk factor is reversible, and the duration of the treatment for the various number of ways that VTE presents amongst different subgroups of patients is yet to be standardized. The controversy is further exacerbated by the numerous different subgroups of patients that undergo different types of VTE, and new data being released on the efficacy of NOACs and DOACs relative to standard and traditional anticoagulation therapy involving VKAs. Whether to extend three-month anticoagulant therapy or stop is decided on a case by case basis, evaluating the risk for increased bleeding against the risk of recurrent thrombosis and VTE [8]. If the choice is made to extend treatment, it is recommended by the American College of Chest Physicians to stay with the anticoagulant therapy, whether it is VKA or a NOAC, rather than changing.

The following systematic review highlights empirical studies and research covering different time courses of anticoagulation treatment for VTE: less than three months, between three and 12 months, and more than 12 months (up to an indefinite period of time). The different factors that affect clinical decision-making regarding anticoagulation therapy and its duration such as idiosyncratic patient subgroups and unique pathophysiological circumstances are also discussed.

## Review

Methods

For the remainder of this review, anticoagulation treatment in the acute period following an initial VTE will be denoted as the initial anticoagulation phase. Anticoagulation administered for up to three months after the initial anticoagulation phase or earlier will be noted as finite anticoagulation. The phrase long-term anticoagulation will be used to denote treatment that extends beyond three months up to six or 12 months. Anticoagulation without a stop date beyond 12 months is deemed as extended/indefinite anticoagulation.

**Figure 1:**
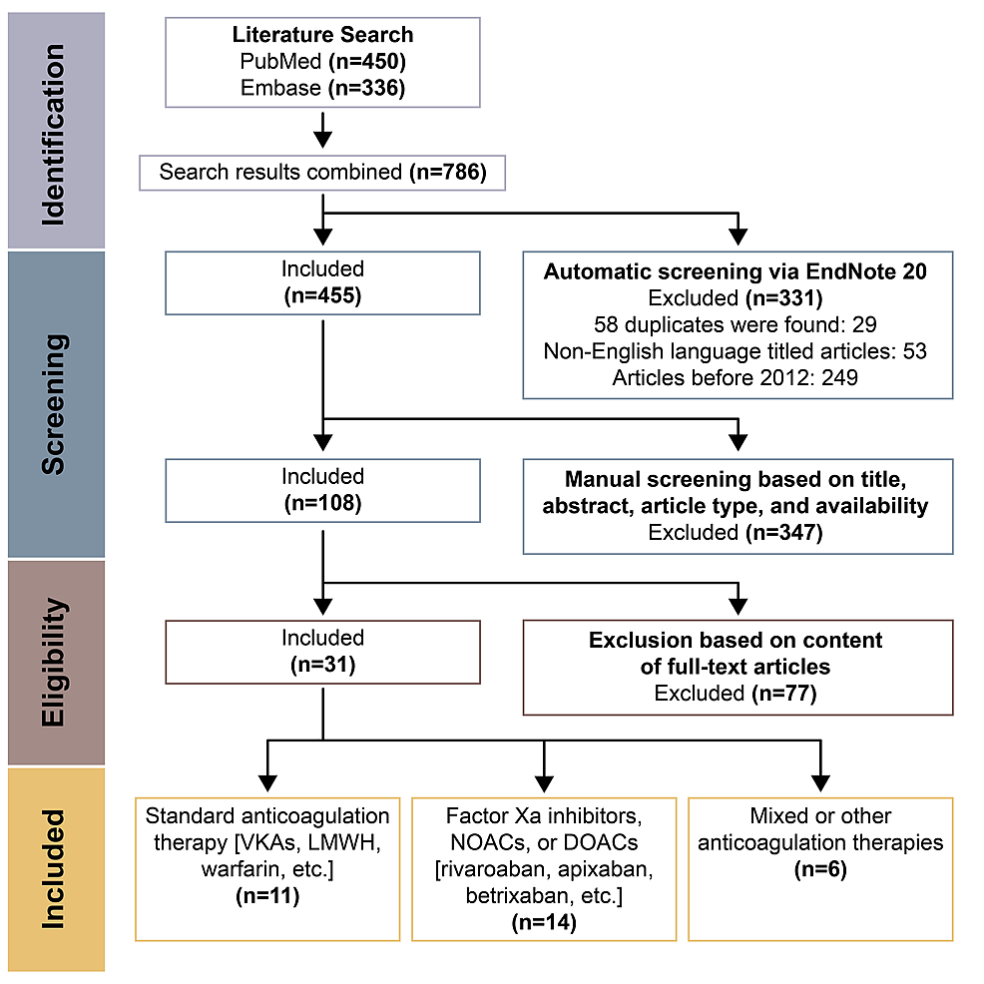
PRISMA flowchart describing the literature screening of studies concerning the duration of anticoagulation in VTE. Screening of the literature has been done mirroring the protocol described in the PRISMA statement [9]. Abbreviations: VTE, venous thromboembolism; VKAs, vitamin K antagonists; LMWH, low molecular weight heparin; NOACs, non-vitamin K antagonist oral anticoagulants or novel oral anticoagulants; DOACs, direct oral anticoagulants; PRISMA, Preferred Reporting Items for Systematic Reviews and Meta-Analyses

This systematic review strictly follows the Preferred Reporting Items for Systematic Reviews and Meta-Analyses (PRISMA) guidelines [9]. A search of the literature was done on June 3rd, 2021, through the databases PubMed, PubMed Central, Medical Literature Analysis, Retrieval System Online (MEDLINE) and Embase. The following query was used on both online databases: ((provoked OR unprovoked OR idiopathic OR distal OR proximal OR transient OR persistent OR reversible OR irreversible) AND (venous thromboembolism OR deep vein thrombosis OR pulmonary embolism)) AND ((anticoagulant OR thrombolytic) AND (treatment OR therapy)) AND ((weeks OR months OR years OR indefinite OR prolonged OR extended OR continuing OR discontinuing OR short term OR long term) AND (duration OR time course)) AND (mortality OR morbidity OR risk of recurrence). In particular, the search through the Embase database was filtered to retrieve only from the Embase database individually (and not to return additional results from MEDLINE or Embase Classic). Regardless of our best efforts and a comprehensive search, we acknowledge that not all relevant research and studies may have been included in this review and some articles may be inadvertently omitted. Seven hundred eighty-six total publications were found and screened. Inclusion and exclusion criteria were applied and only relevant research regarding our research question were considered. A total of 31 articles were kept (Figure 1) [10-40].

Inclusion Criteria

Study selection included the following criteria: studies written in English and conducted on humans, in the last nine years (articles published between 2012 to 2021 were included), that were relevant to our topic and research question (the use and duration of anticoagulation in the treatment of the many types of VTE), peer-reviewed, full texts, including these study types: case reports and studies, clinical trials, observation studies (case-control, cohort, and cross-sectional studies). 

Exclusion Criteria

Duplicates of articles, articles not written in English, books and book chapters, letters to the editor, opinionated articles, editorials, or letters, and in vitro or animal studies were excluded from the literature review. Articles that were strictly abstracts or poster presentations were excluded. Narrative and systematic reviews, meta-analyses, and other literature reviews were excluded. Articles discussing the use of anticoagulation for the treatment of a disease other than primary or secondary VTE were excluded. This process is summarized in Figure 1.

Results

In total, 786 publications were found, 450 from PubMed and 336 from Embase. These publications were consolidated in EndNote 20. Fifty-eight duplicates were found after an initial filter, and 29 articles were removed, giving a total of 757 articles. Fifty-three articles with titles written in a non-English language were removed from consideration, yielding 704 publications. The earliest publication was from 1972 and the most recent was from 2021. Two hundred forty-nine publications before 2012 were not considered and 455 articles were considered for manual screening. Articles were then manually screened based on title, abstract content, article type, and availability, leaving 108 articles to be checked for eligibility. Given that only recently have studies been published focusing specifically on optimizing the duration of anticoagulation therapy, papers that may not have focused on the duration of anticoagulation as a primary outcome were also included.

A large number of the included articles involved analysis of the studies or sub-studies of the following randomized clinical trials: PADIS-PE, PADIS-DVT, APEX, XALIA, RE-COVER, RE-COVER II, Hokusai-VTE, Hokusai-VTE Cancer, Einstein Choice, Einstein-Extension, PEGeD, ExACT, and VISTA [15-17,20,28,29,36,37,39-44]. One case report from India was included that discussed the use of anticoagulants in a female patient with DVT [24]. Many articles featured a discussion on the use of D-dimer testing as part of the algorithm in deciding anticoagulation duration, and four of those articles were included in this review [11,12,23,37]. Ultimately, 31 articles were included in this review of which were primarily focused on standard anticoagulation therapies using VKAs, LMWH, warfarin, etc. and another 14 were primarily focused on factor Xa inhibitors, NOACs, or DOACs; six other articles were using mixed therapies or therapies not previously mentioned [10-40].

Discussion

Options for Anticoagulation

Because this review focuses mainly on the duration of therapy, only a cursory examination of the mechanisms of action and the adverse effects of these drugs will be given. In the initial acute phase of anticoagulation, heparin and VKAs are given. LMWH, UFH, and other heparin-based medications work by directly inhibiting antithrombin III (AT), leading to indirect inhibition of thrombin and clotting factor Xa. Most anticoagulation therapies affect either directly or indirectly, the coagulation cascade, and affect the body’s ability to create fibrin. VKAs like warfarin directly inhibit vitamin K epoxide reductase indirectly, decreasing the clotting ability of clotting factors II, VII, IX, and X. However, its main drawbacks are the medication-food interactions and the need for frequent monitoring of blood drug concentrations. Furthermore, since warfarin is mostly processed by CYP2C9, drug interaction is an additional major concern [45]. The initial treatment of heparin and VKAs is usually staggered, or given as a bridge therapy, with heparin given first for a week and then VKAs initiated seven to 14 days after the VTE event. More recently, the initiation of VKA therapy within one to two days of UFH has evidence of reducing days spent in hospital [2]. Both heparin and VKAs like warfarin can be extended for up to three months but are rarely ever continued into extended treatment, given that DOACs are often the drug of choice for extended timelines. Heparin is often avoided beyond the acute phase of treatment as there is risk for heparin-induced thrombocytopenia (HIT) and an increased risk for osteoporosis. HIT is a severe antibody-related response that causes aberrant and irreversible platelet aggregation, which may lead to thromboembolic events and death, a complication that can result weeks post heparin therapy treatment conclusion [45]. Nevertheless, other preparations of heparin such as LMWH and fondaparinux have been shown to have reduced risk of these adverse effects but are unfortunately not as effectively neutralized by protamine sulfate, an antidote for heparin [46]. Warfarin is often avoided in the extended anticoagulation phase as well, as with all anticoagulant medication due to its increased bleeding risk at its standard efficacious dosage. Nevertheless, in certain patient groups where indefinite anticoagulation is warranted (and prior to NOACs becoming the drug of choice for extended durations), warfarin has been proven efficacious in reducing recurrent VTEs and discontinuation of warfarin after long durations have shown increased recurrence of VTEs; the patients who did continue warfarin for longer periods also suffered from several major and minor bleeding events, compared to those who discontinued warfarin treatment early [15]. Unlike DOACs, reducing the dosage of warfarin does not always decrease the risk of bleeding, and lower-dosage preparations of warfarin have also proven to be significantly less efficacious at preventing recurrent VTE and is a topic of debate [45]. Direct inhibitors, however, are considerably more costly than heparins and warfarin and, unlike heparin and warfarin, have no known antidote; protamine sulfate neutralizes heparins, and vitamin K provides an antidote for VKAs [45]. While warfarin and direct inhibitors are taken orally, heparins are administered intravenously (UFH) or subcutaneously (LMWH and ultra-low-molecular-weight-heparin (ULMWH)) and may cause issues with compliance. Direct inhibitors do not yet have enough safety and effectiveness evidence for long-term usage in certain patient subgroups such as pregnant women and patients with artificial heart valves. As a result, heparins and warfarin still remain the preferred agents for those subgroups.

Some physicians have used an inferior vena cava (IVC) filter alongside anticoagulant therapy in acute VTE to reduce the recurrence of PEs and reduce mortality. Recent evidence suggests that the placement of an IVC filter for three months does not reduce the recurrence of PEs or fatal PE [47]. However, the evidence in the literature is not completely convincing, and IVC filter placement may be beneficial in severe PE cases [5]. Invasive treatments like IVC filter and catheter-directed thrombolysis (CDT) are often reserved for extremely high risk patients and/or for patients with contraindication for anticoagulation. Treatment with CDT in this patient population has been shown to support a reduction in the development of post thrombotic syndrome (PTS), which is cost-effective but associated with an increased risk of bleeding [48,49]. In the past, graduated compression socks were used to combat the development of PTS and associated symptoms. However, more recent studies provide little evidence for graduated compression socks effectively reducing PTS or providing relief of leg pain in acute proximal DVT [50]. In cases where PTS has already developed, trail compression socks may relieve acute and chronic symptoms.

Warfarin was the sole clinically used oral anticoagulant for extended and indefinite durations until recently with the introduction of DOACs. DOACs like the factor Xa inhibitors or “xabans” work by inhibiting factor Xa directly, inhibiting thrombin and fibrin formation. Xaban DOACs are quickly becoming the drug of choice for anticoagulation across many patient subgroups and for many timelines. Extended DOAC therapy after an initial anticoagulation course was shown to prevent recurrent VTE in patients with an initial unprovoked VTE. DOACs like rivaroxaban have been shown to decrease recurrent VTE in both unprovoked and provoked populations, but are ideal for those that are affected by unprovoked VTEs. For those with transient risk factors, for example VTEs provoked by major surgeries or traumas, DOACs are recommended but not for extended durations. Ultimately, DOACs reduce recurrent VTEs without increasing the risk of bleeding relative to standard anticoagulation therapies like warfarin or aspirin [8]. Risk of bleeding has been shown to be high, however, in those with provoked VTE due to a major persistent factor, such as cancer, additionally, more data is required as bleeding is seen across the spectrum of anticoagulation given and not just limited to DOACs for cancer-related VTEs. Lower intensity regimens for DOACs like rivaroxaban and apixaban are available for those with persistent risk factors and are preferred over warfarin and aspirin [51].

Recently, direct thrombin inhibitors (DTIs) have also been introduced as a DOAC form of anticoagulation therapy. Both parenteral and oral direct thrombin inhibitors have been studied for the prevention and treatment of venous thromboembolism (VTE), the prevention of thromboembolic complications in patients with HIT or at risk for HIT who are undergoing percutaneous coronary intervention (PCI), acute coronary syndromes (ACS) with and without percutaneous transluminal coronary angioplasty (PTCA), secondary prevention of VTE, and secondary prevention of VTE [52]. Common DTI DOACs are lepirudin, desirudin, bivalirudin, dabigatran, and argatroban. The RE-COVER trials have also shown that dabigatran is non-inferior to warfarin in terms of safety and efficacy, but fewer overall bleeding events were observed with warfarin [19,41,42,52].

The role of aspirin (ASA) in VTE treatment is limited due to anticoagulation therapy’s effectiveness at reducing recurrent VTE [53,54]. At low doses, aspirin is used as an anticoagulant by limiting platelet aggregation due to its irreversible inhibition of thromboxane. However, ASA does serve a role in specific cases where indefinite anticoagulant therapy is recommended but declined by the patient (either due to cost of DOACs or noncompliance with warfarin or heparin).

Duration of Treatment

Initial phase: finite anticoagulation for three months: Finite anticoagulation of about three months after VTE is mainly recommended for patient subgroups with a low risk of recurrence of VTE or an extremely high risk of bleeding. Namely, patients with an initial iatrogenic VTE, provoked by a major risk factor such as a major surgery or a major trauma are indicated for this duration. Moreover, increasing the duration beyond three to six months of administration might do more harm than good and it is suggested that progressing a patient in this category to an indefinite duration only be considered when the major transient risk factor recategorizes the patient as someone who is now prothrombotic [55]. This is not to say however, that increasing anticoagulation duration beyond three months is always harmful, especially if anticoagulants like rivaroxaban are used, as seen in Khorana et al., 2017 [22]. Rivaroxaban has been shown to decrease rates of recurrent VTE with extended anticoagulation without increasing rates of bleeding.

Patients who are at increased risk of bleeding should also avoid extended and indefinite anticoagulation. For example in Iñurrieta et al. 2018, although some patients >75 years of age had suffered from an unprovoked VTE, which almost always warrants an extended duration of anticoagulation, their increased risk of major bleeding events and severe hemorrhages benefited from an early discontinuation of therapy. This discontinuation was also helpful for those who suffered anemia [30].

Unfortunately, there is an overarching issue with studying different anticoagulation durations in the provoked VTE group as they are not always clinically interesting and do not present scientific or financial incentives to the pharmaceutical industry. Most provoked VTE cases with major transient risk factors that warrant finite anticoagulation are simply patients who suffered a VTE due to a major surgery or trauma. When considering the fact that the rate of recurrence of VTE is low in the bulk of these patients and that administering anticoagulants might do more harm than good (where the primary outcome of anticoagulants are to prevent recurrent VTEs), extended durations for this group are rarely ever observed. However, if such a patient were to suffer from a recurrent VTE, it is likely that discontinuation of anticoagulation would be to blame for the reoccurrence and not a possible miscategorization or mis-stratification of the patient and their risk factors, namely because patient groups and risk factors for anticoagulation are somewhat arbitrary and non-rigorous. This causality dilemma is also a contributing factor to the controversy surrounding the duration of anticoagulation and also why physicians tend to administer anticoagulants for longer periods than suggested. Luckily, this does incentivize the discovery and testing of medicines that are safer to administer for longer durations regardless of patient subgroup and likely explains the rise in popularity of DOACs.

Long-term phase: beyond three months up to six and 12 months: As the debate of whether to discontinue or continue anticoagulation therapy resumes, studies supporting opposing sides continue to emerge. That being said, the more popular and prominent finding is that continued anticoagulation therapy has proven over and over again that it significantly reduces the risk for recurrent DVT and VTE events even at the cost of increasing the risk of major bleeding. The following mentioned studies delve deeper into the prospects of continuing anticoagulation therapy beyond an index point of three months, up to 12 months of treatment. A study conducted on the duration of anticoagulant therapy after a second episode of VTE revealed a substantial difference between the chances of undergoing a recurrent VTE in patients with continued therapy versus those with a discontinued one. In patients that were treated for six months only, the study revealed a VTE recurrence rate of 21% after a four-year continuous follow up versus a rate of 2.6% in the patients that were placed on a continued anticoagulant regimen [relative risk 8.0; 95% confidence interval (CI): 2.5-26]. It must be noted, however, that those who had a discontinued anticoagulation therapy suffered less reported incidents of major bleeding throughout the trial (2.7% versus 8.6%; relative risk 0.3; 95% CI: 0.1-1.1) [56].

In 2015, a similar study with a follow up time of one year was conducted to corroborate these results and prove that even though the risk of major bleeding increases with continued treatment, it is still significantly less than the risk of a recurrent VTE. In the cohort of patients with discontinued treatment, a VTE recurrence incidence rate was measured to be 9.4/100 patient-years with a cumulative rate of 15% after one year and rose up to 33% after a five-year follow up. This risk surpassed that of major bleeding incidents in the cohort of patients with continued therapies: 2.4/100 patient-years [14]. In another double-blind trial, patients with unprovoked proximal deep-vein thrombosis that were initially placed on six months of oral anticoagulant treatments were split into two cohorts. The first cohort continued treatment with 18 months of warfarin therapy whereas the second cohort were given a placebo treatment instead. During the 18 months of warfarin treatment, there was no recorded incidence of VTE recurrence nor major bleeding amongst the 50 patients in that cohort, where as in the cohort taking the placebo, 16 out of 54 patients reported either a recurrence or a major bleeding leading to a cumulative risk of 29.6% (hazard ratio, 0.03; 95% confidence interval: 0.01 to 0.09; P<0.001). After the 18 months, treatments were ceased for both cohorts and the patients statuses were being followed up with for a cumulative of a 42-month study period. Results showed that when the warfarin treatment was stopped, an incidence rate in that cohort rose up to 14 patients (cumulative 36.8%) and was very similar to those placed on the placebo treatment which reported incidents in 17 patients (cumulative risk, 31.5%). The study's conclusion demonstrates the benefits of continued anticoagulation treatment as well as the lack thereof once treatment is discontinued [36].

In another study involving 7,469 patients, the risk of recurrence of VTE in patients with continued (>three months) versus discontinued (<three months) treatments were compared to the risk of major bleeding for each. As expected the continued anticoagulation treatment proved to substantially reduce the risk of VTE recurrence after an additional three months (0.70% vs 1.70%), six months (1.41% vs 2.34%), nine months (1.82% vs 3.01%), and 12 months (1.97% vs 3.01%) of treatment (all, p < 0.05). However, what is particularly interesting is that the risk of major bleeding observed amongst patients with continued rivaroxaban therapy versus discontinued therapy was not statistically significant [22].

Extended phase: indefinite or lifelong anticoagulant treatment: In the clinical management of VTE, the critical decision of whether or not to extend anticoagulant treatment following the initial period to prevent recurrent VTE must be addressed. To give some perspective to the patient type and the rate at which extended therapy is initiated for greater than 12 months, the XALIA study conducted by Angeno et al., 2016, reported 55% of patients with a first time unprovoked VTE, 42% of patients with a VTE due to provoked or transient risk factors, and 43% of patients with VTE due to cancer received anticoagulant therapy for longer than 12 months [17]. Generally, physicians consider the risk of VTE recurrence over the risk of major bleeding events in determining whether the extension of anticoagulant treatment is appropriate. It must be noted, in the previous study, the rate of fatal bleeds outpaced that of fatal PE in all three subgroups, where two-thirds of major bleeds and one-third of fatal bleeds occurred after the three-month initial period. Thus, the risk of a major bleed cannot be underestimated after the three-month initial treatment of anticoagulants. However, the study suggests there is no significant difference in major bleeding events. The decision to extend treatment is more complex than this, especially in unprovoked VTE patients.

The common factors physicians used for corroborating a 12 month or indefinite anticoagulant treatment decision were age (older than 65 years), an unprovoked causal factor, proximal VTE over distal and recurrent VTE or residual thrombus [10,38]. Few studies included the use of D-dimer level monitoring in conjunction, and results were similar to those who did not, suggesting D-dimer may not provide pertinent evidence in the decision-making process [11]. However, in a large retrospective claims database analysis done by Coleman et al., 2019, (greater than three-month treatment with rivaroxaban) and statistical analysis of the EINSTEIN EXTENSION and EINSTEIN CHOICE by Prins et al., 2018, (one year or more rivaroxaban treatment) reported a recurrent VTE risk reduction of 83% and 75% in provoked VTE patients with persistent risk factors and minor transient risk factors respectively [34,31], suggesting a subset of provoked VTE patients may benefit from extended rivaroxaban treatment.

The introduction of DOACs, like rivaroxaban, have provided some promising results for a non-inferior, more convenient alternative anticoagulant regimen to prevent recurrent VTE. In a multicenter prospective registry study done by Antonucci et al. in 2020, the most frequently prescribed treatment for extended treatment (anticoagulation therapy greater than six months up to a year) were apixaban 33.8% (5mg BID), rivaroxaban 29.3% (20mg OID), apixaban 13.1% (2.5mg BID), edoxaban 12.6% (60mg OID), LMWH/fondaparinux 6.5%, VKA 3.0%, dabigatran 1.5% (150mg BID); this study also suggests that physicians are aware of and are more comfortable with DOACs and administer them in extended anticoagulant treatment to reduce recurrent VTEs [38]. Rivaroxaban is the most studied of the DOAC drugs. In the XALIA study comparing rivaroxaban to standard anticoagulant treatment (LMWH/fondaparinux followed by or overlapped with VKA) with a mean duration of 181 days (94-310) for rivaroxaban and 190 (97-368) for standard treatment [10]. The study reported a lower frequency of major bleeding and no fatal bleeds in the rivaroxaban group compared to two in the standard anticoagulant treatment group. Additionally, the rivaroxaban group had less frequent recurrent VTEs and all-cause mortality compared to standard anticoagulant treatment. Rivaroxaban showed to be an effective single-day treatment associated with shorter hospital days and simplified management. The continued use of rivaroxaban beyond three and six months may be the best line of treatment to prevent recurrent VTE displayed in retrospective U.S. claims database studies done by Khorana et al., 2017 [22]. The study showed a recurrent VTE relative risk reduction of 45% at 12-month follow-up when comparing continued rivaroxaban treatment (mean of 341 days) to six months of rivaroxaban treatment with no statistically significant difference in risk of major bleeding events. Similarly, Berger et al., 2017, reported a 65% relative risk reduction in recurrent VTE (pulmonary embolism was significant, recurrent DVT rate was low but not statistically significant) at 12-month follow-up of continued rivaroxaban treatment (mean of 247 days) compared to six-month treatment with no significant difference in risk of a major bleeding event [26]. The standard anticoagulation therapies and the most recommended anticoagulation therapies and the therapies that this review focuses on are summarized in Table 1 and Table 2.

**Table 1:** Studies on the use of various anticoagulation therapies for treating VTE and other related diseases over various durations. Table showing the studies on the use of differing durations of various anticoagulation therapies for various ailments from the past seven years. Studies are binned into the type of therapy administered: some studies primarily focused on standard anticoagulation therapies such as VKAs, LMWH, warfarin, etc., while others focused on factor Xa inhibitors, NOACs, or DOACs. Studies that could not have been neatly binned into one group or the other, or had used treatments not belonging to either group were binned into their own category. LMWH, low molecular weight heparin); VKAs, vitamin K antagonist; VTE, venous thromboembolism; IQR, interquartile range; PE, pulmonary embolism; DVT, deep vein thrombosis; MM, multiple myeloma; EPO, erythropoietin; Hb, hemoglobin; IDDVT, isolated distal deep vein thrombosis; DOAC, direct-acting oral anticoagulants; US, United States; PDVT, proximal deep vein thrombosis; PTS, post-thrombotic syndrome; VPM, Vienna Prediction Model; C-PTP, clinical pretest probability of pulmonary embolism; RI, renal impairment; CrCl, creatinine clearance; OAC, oral anticoagulant.

Author	Country	Study Population & Duration	Findings and Conclusions
Ageno et al., 2015 [10]	Spain, Italy, France, Israel,Greece, Switzerland, Czech Republic, and Macedonia	Patients with VTE (n=6944): unprovoked (n=2851), provoked or transient risk factors (n=2209), cancer (n=1884); 2 years	In patients with unprovoked VTE, major bleeding and VTE recurrence were comparable, but in those with transitory risk factors or malignancy, major bleeding outpaced VTE recurrence. Most importantly, in all three patient groups, fatal bleeds outnumbered fatal PE recurrences. The risk of bleeding after three months of therapy should not be underestimated. After three months of anticoagulant therapy, the risk of recurrence is low in patients with removable risk factors. Patients with spontaneous VTE and malignancy are most at risk. The risk of recurrence after stopping therapy should be weighed against the risk of bleeding if therapy is continued.
Kearon et al., 2015 [11]	Canada	Patients with symptomatic unprovoked proximal deep venous thrombosis of the legs or pulmonary embolism who had completed 3 to 7 months of uninterrupted warfarin (n=410); 6 months	Patients with spontaneous proximal DVT or PE who had a negative D-dimer test during anticoagulant treatment and 1 month later had recurrent VTE. D-dimer testing was positive in less than 5% of anticoagulant patients, indicating that skipping this test and testing only after anticoagulant withdrawal is safe. The use of D-dimer testing to determine therapy duration in unprovoked VTE remains controversial.
Lee et al., 2015 [12]	Korea	Patients with VTE (acute symptomatic proximal DVT, PE, or both) originally undergoing chemotherapy for treatment of multiple myeloma (n=48); <6 months	To accurately predict recurrence of VTE, patients at high risk of recurrence may require prolonged anticoagulant therapy. Dalteparin can be used to treat VTE in MM patients. In addition, higher hemoglobin levels and concurrent EPO use were linked to increased risk of recurrence, recommending cautious use until VTE is fully cured. As a surrogate for VTE in MM patients, D-dimer levels changed significantly before and after dalteparin therapy.
Li et al., 2015 [13]	New Zealand	Patients with axial IDDVT (n=507); 3-12 months	In the absence of malignancy, 6 weeks of anticoagulation seems to be effective and safe, with low rates of proximal propagation and VTE recurrence. Because proximal VTE propagation and recurrence are rare in this patient group, but anticoagulation-induced bleeding is common, the benefits and risks of anticoagulation vary between isolated distal DVT and proximal DVT.
van der Hulle et al., 2015 [14]	Netherlands	Patients with late second VTE (n=131); 3, 6, and 12 months, and indefinitely	However, selecting patients with low VTE recurrence risk based on the time between anticoagulant discontinuation and the second VTE is not acceptable. Its risk-benefit ratio must be evaluated case by case.
Couturaud et al., 2015 [15]	France (PADIS-PE study)	Patients who had experienced a first episode of symptomatic unprovoked pulmonary embolism (i.e., with no major risk factor for thrombosis) and had been treated initially for 6 uninterrupted months with a vitamin K antagonist (n=371); 2 years	In patients with a first episode of spontaneous pulmonary embolism, 18 months of warfarin therapy reduced the composite outcome of recurrent venous thrombosis and severe hemorrhage compared to placebo. After stopping the anticoagulant, the effect faded.
Cohen et al., 2016 [16]	400 sites in North America, Europe, South America, South Africa, Asia, and Australia (APEX study)	Patients with acute heart failure, respiratory failure, infectious disease, rheumatic disease, or ischemic stroke and at increased risk for VTE (n=3759): cohort 1 (patients who had positive D-dimer results n=2314) and cohort 2 (patients who had positive D-dimer results or 75 years or older n=3407); <3 months	It had fewer thromboembolism risks than enoxaparin. Betrixaban reduced major and fatal bleeding. Extended-duration betrixaban caused more nonmajor hemorrhage than standard-duration enoxaparin, but not more major bleeding. Intracranial hemorrhage was uncommon in both groups, though less so with betrixaban. But betrixaban reduced fatal and non-fatal pulmonary embolisms and mortality in critically ill patients.
Ageno et al., 2016 [17]	21 European countries (XALIA study)	Patients with DVT (n=4768); 6 months	Rivaroxaban had a lower risk profile (less severe bleeding and recurrent VTE) than other anticoagulants across a wide range of patient demographics. Rivaroxaban was associated with fewer hospitalizations and a shorter least-squares mean stay than conventional anticoagulant therapy.
Raskob et al., 2016 [18]	28 countries worldwide	Patients with VTE (n=5365): DVT (n=3532), PE (n=1359), PE with DVT (n=477); 6 months	The all-oral apixaban dosage schedule caused no recurrences or severe bleeding. Apixaban-treated patients had less early significant bleeding than those treated with conventional therapy at 7, 21, and 90 days. Apixaban alone (no enoxaparin first) was effective for VTE and is similar to rivaroxaban.
Klok et al., 2016 [19]	31 countries worldwide (RE-COVER [41] and RE-COVER II [42] substudy)	Patients with symptomatic and confirmed proximal deep vein thrombosis (DVT) or acute pulmonary embolism (PE) (n=5107); 6 months	After 3–6 months, the VTE-BLEED score may help determine if anticoagulation should be continued. If you are using dabigatran or warfarin, the VTE-BLEED score identified 25% of patients with increased bleeding risk. Dabigatran (or another target-specific oral anticoagulant)-associated bleeding risk factors may differ from warfarin-associated bleeding risk factors. It is more common in the first few days or weeks of oral anticoagulation, especially for vitamin K antagonists, but also for other anticoagulants. For anticoagulant therapy beyond 3 or 6 months, the patient's "chronic" bleeding risk must be assessed.
Verhamme et al., 2016 [20]	37 countries worldwide (Hokusai-VTE study)	Patients with VTE (n=8240): confirmed acute, symptomatic DVT that involved the popliteal, femoral, or iliac veins, or acute (n=4921), symptomatic PE with or without DVT (n=3319); 3-12 months	Patients with creatinine clearance 30-50 ml/min, weight 60 kg or less, or taking verapamil or quinidine had half the excess drug exposure. Despite the lower median pre- and post-dose edoxaban levels (30 mg vs. 60 mg), effectiveness and safety were maintained. Thus, doctors and patients can rest assured that monitoring anticoagulant action or medication levels is not necessary. A higher incidence of bleeding was found in those who met the dosage reduction criteria, suggesting that these patients are at risk and could benefit from safer treatment. In patients who could reduce their dose, edoxaban bled less than warfarin. A large proportion of the study's participants meet the dosage reduction criterion. Older, female, and Asian patients had widespread and severe VTE.
Donadini et al., 2017 [21]	Italy	Patients with IDDVT (n=321); <3 months	Those with spontaneous or prior VTE have a high risk of recurrent VTE after IDDVT treatment. After 3.5 years, 25.3% of IDDVT patients had recurrent VTE. Half of the incidents occurred in the first year, with a total incidence of 13%. 15% after 1 year, 26% after 3 years. A 4- to 6-week anticoagulant treatment may be sufficient for IDDVT with transitory risk factors.
Khorana et al., 2017 [22]	US	Patients with an initial VTE (n=11452); <12 months	Long-term treatment reduced the risk of recurrent VTE by 35% (12-month rate, 1.97 vs 3.01%) without affecting severe bleeding. Taking rivaroxaban for 6 months reduced VTE recurrence by 54%. (Recurrent VTE, 1.72 vs 3.70%). There were no statistically significant differences in the risk of serious bleeding between the two groups at 3 or 6 months. The 12-month bleed rates were similar in both groups. Extended rivaroxaban therapy may reduce VTE recurrence without affecting bleeding risk.
Anniccherico-Sanchez et al., 2017 [23]	Spain	Patients with PE and who have completed greater than or equal to 3 months of anticoagulation (n=477); 3 months or indefinitely	Pneumoembolism recurrence is high. Patients with idiopathic pulmonary embolism, a high thrombotic load, and persistently elevated D-dimer levels should be evaluated for permanent anticoagulant therapy.
Babu et al., 2017 [24]	India	Patient (64 year old female homemaker) with DVT (n=1); 6 months	A repeat color Doppler ultrasonography of her neck revealed a 90% reduction in DVT after 6 months of anticoagulant therapy. A 6-month CT scan revealed dissolution of the lesion and reexpansion of the right middle lobe, with a 12-month CT scan showing similar results.
Gibson et al., 2017 [25]	400 sites in North America, Europe, South America, South Africa, Asia, and Australia (APEX [16] substudy)	Patients with acute heart failure, respiratory failure, infectious disease, rheumatic disease, or ischemic stroke and at increased risk for VTE (n=3759): cohort 1 (patients who had positive D-dimer results n=2314) and cohort 2 (patients who had positive D-dimer results or 75 years or older n=3407); <3 months	Treixaban's extended-duration enoxaparin (6-14 days) reduces fatal or irreversible events. The benefit continues during active treatment (35–42 days) and follow-up (77 days). The full dose (80 mg) of betrixaban reduced fatal or irreversible events, but the modified dose (40 mg) did not.
Berger et al., 2017 [26]	US	Patients with unprovoked VTE (n=4814); 3 months or indefinitely	Withdrawal after 3 months reduced the risk of VTE recurrence by 44% (12.45 vs. 2.60 ). Recurrent VTE was reduced by 66% in patients who took rivaroxaban for six months or longer (from 4.62 percent vs 1.64 percent for discontinued vs continued cohorts). At 3 or 6 months, there was no significant difference in major bleeding risk between the two groups who continued. Comparing the two groups, the continuing group had lower risk of recurrent PE.
Goldhaber et al., 2017 [27]	31 countries worldwide (RE-COVER [41] and RE-COVER II [42] substudy)	Patients with symptomatic and confirmed proximal deep vein thrombosis (DVT) or acute pulmonary embolism (PE) (n=5107); 6 months	Dabigatran reduced VTE/VTE-related mortality in the RE-COVER studies, but increased bleeding events with age or renal function. Dabigatran is mostly eliminated via the kidneys, and RI patients have higher plasma dabigatran concentrations. Age, RI, or both increased bleeding risk with dabigatran and warfarin. Dabigatran bled less than warfarin in most subgroups. Dabigatran had no effect on VTE recurrence or bleeding in the RE-COVER subgroups based on age and renal function. Those with acute VTE who took dabigatran had lower rates of recurrent VTE (due to higher concentration of dabigatran) and higher rates of bleeding. The incidence of recurrent VTE with dabigatran and warfarin was comparable.
Weitz et al., 2017 [28]	267 countries worldwide (EinsteinChoice study)	Patients with confirmed symptomatic DVT and/or PE who have completed 6-12 months of prior anticoagulation treatment; 6-12 months	Rivaroxaban (20 mg) was found to be more effective than aspirin in preventing recurrent venous thromboembolism in patients who were undecided about anticoagulation. The reduced risk of recurrence was linked to a rate of bleeding comparable to aspirin.
Raskob et al., 2018 [29]	13 countries (Hokusai VTE Cancer study)	Patients with cancer and incident DVT or acute symptomatic PE (n=1050); 12 months	For up to 12 months, subcutaneous dalteparin was noninferior to recurrent venous thromboembolism or severe bleeding. Edoxaban had a lower rate of recurrent symptomatic DVT than dalteparin. Edoxaban caused more major bleeding than dalteparin. This was due to edoxaban's increased risk of upper GI hemorrhage.
Iñurrieta et al., 2018 [30]	Spain	Patients aged >75 years with a first episode of unprovoked PE or proximal DVT (n=7,830): PE with or without concomitant DVT (n=5058), proximal DVT alone (n=2772); 6 and 12 months or indefinitely	After the third month of treatment, major bleeding was more common than PE recurrences (101 vs. 44), and fatal bleeding was more common than PE (19 vs. 3 deaths). Long-term VKA or LMWH users had more frequent and severe bleeding. It is important to consider the clinical significance of bleeding beyond the first 3 months even in patients who did not bleed. Severe bleeding in the first three months quadrupled the risk of rebleeding. DVT patients had a lower risk of PE recurrence than PE patients. Patients over 75 with unprovoked DVT or anemia may benefit from early treatment cessation (3 months).
Prins et al., 2018 [31]	267 countries worldwide (EinsteinChoice [28] substudy) and 322 countries worldwide (Einstein-Extension [44] substudy)	Patients with symptomatic proximal DVT or PE (n=4,553): EINSTEIN CHOICE trial (n=3365) and EINSTEIN EXTENSION trial (n=1188); 6-12 months	A substantial transient risk factor such as major surgery or trauma caused most unprovoked VTE cases to recur. A substantial transient risk factor for VTE does not warrant prolonged anticoagulation. Discontinuing anticoagulant therapy in patients with VTE caused by minor chronic or transient risk factors increases the risk of recurrence. Compared to placebo, rivaroxaban reduced recurrent VTE by 0.5%. Extended anticoagulation is helpful for VTE caused by significant transient risk factors Minor chronic or transient risk factors can cause VTE. It reduced the risk of recurrence in both unprovoked and provoked VTE and had a similar bleeding rate as aspirin or placebo. As an extended VTE treatment, rivaroxaban's ease of use and dose reduction from 20mg to 10mg once daily make it an
Yamashita et al., 2018 [32]	Japan	Patients with acute symptomatic VTE (n=3027): transient risk group (n=855), unprovoked group (n=1477), cancer group (n=695); 12 months	The study's main findings were: In VTE patients with a transient risk factor, longer-term anticoagulation therapy beyond 3 months was frequently used, contrary to current guidelines, and there was no difference in cumulative incidence of recurrent VTE beyond 1 year regardless of anticoagulation status; (2) In unprovoked VTE patients or those with prior VTE, the risk of recurrent VTE increased with discontinuation of anticoagulation therapy. However, in the current study, only 37.3% of VTE patients with a transient risk factor discontinued anticoagulation therapy, indicating that physicians were more concerned about the risk of recurrent VTE than the risk of bleeding.
Albertsen et al., 2018 [33]	Denmark	Patients with incident VTE (n=73993): unprovoked (n=36009), provoked (n=27966), cancer (n=10018); >6 months	There is no consensus on how long OAC treatment should last for unprovoked VTE, which is associated with a higher risk of recurrence. Unprovoked VTE had an 18% higher (adjusted) relative risk of recurrence than provoked VTE. The probability of recurrence was similar for unprovoked and provoked venous thromboembolism for the first 6 months, but then diverged. Unprovoked VTE had the same 10-year recurrence risk as cancer-related VTE. One reason is rebound thrombosis after stopping OAC treatment for an unprovoked venous thromboembolism. Because of concerns about bleeding, some people refuse extended OAC treatment. The lowest risk group of the three studied had a recurrence rate of 15% after a decade. Cancer-related thromboembolism patients had the highest recurrence risk. After a 10-year follow-up, they discovered that unprovoked and cancer-related venous thromboembolism had similar recurrence rates. Given the wide range of VTE patients, their findings call for reconsidering VTE patient categorization.
Coleman et al., 2019 [34]	US	Patients with provoked VTE (n=4990); 3 months or indefinitely	Remaining on rivaroxaban reduced the risk of repeat VTE by 44% without worsening severe hemorrhage compared to stopping anticoagulants (no anticoagulants or nonaspirin antiplatelet medications but may have taken aspirin). Severe bleeding rates did not differ between groups. Berger et al., 2017 [26] reach a similar conclusion (reduction in recurrent thrombosis without an increased risk of major bleeding). Recurrent VTE may be reduced with long-term rivaroxaban use, with the greatest risk (3.1%) and decrease (83%) in those with a mild risk factor. Those who continued taking rivaroxaban had a 27% higher rate of VTE recurrence and a 40% lower rate of serious bleeding compared to those who stopped. It was found that people with moderate persistent risk factors who took rivaroxaban for 3 months or longer had lower rates of recurrent VT.
Ageno et al., 2019 [35]	Spain, Italy, France, Israel, Greece, Switzerland, Czech Republic, and Macedonia (XALIA [17] substudy)	Patients with IDDVT or PDVT (n=5136): IDDVT (n=1065), PDVT (n=3317); 6 months	IDDVT patients were younger and had less unprovoked VTE than PDVT. Recurrent VTE rates were numerically lower in IDDVT patients than in PDVT patients, but this was not statistically significant after adjusting for baseline characteristics.
Couturaud et al., 2019 [36]	France (PADIS-DVT study)	Patients with symptomatic unprovoked proximal DVT (n=104); > 6 months, <18 months, or indefinitely	18 months of warfarin treatment reduced the risk of recurrent VT and severe bleeding by 97%. This benefit faded after stopping anticoagulation. While there was no rebound effect compared to the placebo group in the first few months, the risk of recurrence increased linearly over the next 24 months, resulting in an annual recurrence rate of 9.4%. Additional randomized studies on unprovoked venous thromboembolism patients show a loss of extended treatment benefit after stopping anticoagulation. Indefinite anticoagulation is recommended for patients with a first episode of unprovoked proximal DVT. Symptomatic deep-vein thrombosis recurred in 90% of patients treated for 6 or 24 months, with 3% (1 of 31) being fatal. Most recurrences were unavoidable, even though they were milder.
Kearon et al., 2019 [37]	Canada (PEGeD study)	Patients with signs and symptoms of PE (n=2017): Low C-PTP (n=1752), Moderate C-PTP (n=218), High C-PTP (n=47); 3 months	Pneumoembolism should be ruled out in patients with C-PTP and D-dimer levels less than 1000 ng/ml. Pneumoembolism is ruled out in patients with C-PTP > 1 and d-dimer > 500 ng/mL. This method reduced the number of chest imaging tests for suspected pulmonary embolism.
Antonucci et al., 2020 [38]	Italy	Patients with VTE (n=472); >3 months, <12 months, or indefinitely	Following a VTE event, the Italian vascular physicians in this study mostly followed the international 3-month AT time. Whether the index episode was secondary or unprovoked, the treating doctors choose to stop or continue AT. There was likely no risk factor identified because they looked at the etiology (secondary or unprovoked) along with other variables like the patient's clinical state and risk factors for bleeding or thrombosis.
Bradbury et al., 2020 [39]	UK (ExACT study)	Patients with a first episode of unprovoked VTE and who have completed greater than or equal to 3 months of anticoagulation (n=273); 3 months, <2 years, or indefinitely	Extended AT reduces unprovoked VTE recurrence risk. Extended AT reduced the incidence of VTE recurrence by 80% after an unprovoked VTE. Due to the small number of events, the extended AT arm had more bleeding episodes. The ExACT trial did not routinely screen for DVT, but all patients had PTS (DVT and PE). However, only a minority of patients had severe PTS after two years, and there was no difference in frequency or severity between patients who received extended vs stopped AT.
Geersing et al., 2020 [40]	Netherlands (VISTA study)	Patients with unprovoked VTE (n=883); <6 months or <12 months	Based on this research, using a risk prediction model to assess recurrence risk in patients with spontaneous VTE did not reduce risk. The VPM predicted recurrent VTE well. The model was well calibrated for low-risk patients (up to 4 percent ). A meta-analysis of individual participant data found that the VPM underestimated recurrence risk in unprovoked VTE patients.

**Table 2:** Common anticoagulation therapies. Table showing the common anticoagulation medications given, their mechanisms of action, common general and brand names, and recommended durations of treatment. Mechanical modes of treatment, such as compression socks, or more invasive methods reserved for higher risk to maximal risk patients such as an inferior vena cava (IVC) filter or catheter-directed thrombolysis were not included. LMWH, low-molecular-weight-heparin; AT, antithrombin III; VKAs, vitamin K antagonists; HIT, heparin-induced thrombocytopenia; DOACs, direct oral anticoagulant; ASA, aspirin; NOACs, novel-oral-anticoagulants or non-vitamin K antagonist-oral anticoagulants; DTI, direct thrombin inhibitors.

Class of anticoagulant	Mechanism of action	Drugs	Common treatment durations
Heparin based products	Directly inhibits antithrombin III (AT) leading to indirect inhibition of thrombin and clotting factor Xa	Heparin, Dalteparin, Enoxaparin, Fondaparinux	LMWH in combination with VKAs are almost always given in the acute initial anticoagulation phase. Heparin is rarely ever considered for extended or indefinite anticoagulation therapy, namely for its risk of heparin-induced thrombocytopenia (HIT), and in some cases, osteoporosis. LMWH also has issues with compliance as it is taken by injection.
Vitamin K Antagonist (VKA)	Directly inhibits vitamin K epoxide reductase indirectly decreasing the clotting ability of clotting factors II, VII, IX, and X	Warfarin	Warfarin is almost always given in the acute initial anticoagulation phase in the first three months of therapy. For extended to indefinite anticoagulation, warfarin is considered inferior to DOACs due to a relative increased risk of bleeding, but can be given if the cost of DOACs impose a barrier to compliance. Also, unlike DOACs, warfarin loses efficacy at lower doses without necessarily being safer.
Antiplatelet	Directly and irreversibly blocks the formation of thromboxane A2 in platelets leading to decreased platelet aggregation	Aspirin (low dose)	Though inferior to warfarin and DOACs for reducing recurrent VTE risk, aspirin can still be given for indefinite anticoagulation when DOACs are declined by patients (due to either the inconvenience associated with repeated testing or the cost).
Direct clotting Xa inhibitors ("xabans"), novel oral anticoagulant or non-vitamin K oral antagonist anticoagulant (NOACs), direct oral anticoagulants (DOACs)	Directly inhibits clotting factor Xa leading to inhibition of thrombin (IIa) and decreased fibrin formation	Apixaban, Betrixaban, Edoxaban, Rivaroxaban	DOACs are the first line treatment and drug of choice for extended anticoagulation for 6-12 months after initial anticoagulation. They are also recommended for indefinite anticoagulation at low doses with frequent reassessments for bleeding and hemorrhage risk when compared to warfarin and aspirin.
Direct thrombin (IIa) inhibitors (DTI), also called direct oral anticoagulants (DOACs)	Directly inhibits the thrombin enzyme (IIa) by binding to the active site only (univalent) or by binding to the active site and an additional exosite (bivalent**)	Argatroban, Dabigatran, Bivalirudin**, Hiruden**	DTIs have not yet been fully studied as treatment options in the extended to indefinite anticoagulation therapy regime, but have been found to be an alternative to warfarin and aspirin. They can also be alternatives to heparin when the risk for HIT is high.

## Conclusions

Anticoagulant therapy beyond the acute phase of a VTE is a difficult decision for physicians. Anticoagulation should be prolonged if the risk of repeat thrombosis is high after the initial three- to six-month period. Some patients may benefit from prolonged anticoagulation, long term or even lifelong, and might vary on a case-by-case basis based on patient-specific risk factors. Physicians and patients should engage in shared decision making and discuss compliance, risks and benefits, and patient preferences when making these decisions. In cases of VTEs provoked by a major risk factor, anticoagulation for three to six months is recommended. Finite anticoagulation is also recommended for those with high bleeding risk and advanced age (>75). In this systematic review, the most common factors that corroborate an extended (12 months or longer) anticoagulant treatment were advanced age (>65 years), unprovoked VTE, proximal VTE, recurrent VTE during treatment, and presence of residual thrombus. Furthermore, VTEs provoked by cancer are also candidates for indefinite anticoagulation, although prognosis of both recurrent thrombosis and bleeding is poor. DOACs, like rivaroxaban, provide a promising alternative to VKA and LMWH/fondaparinux, especially when used for greater than six months. Rivaroxaban was associated with shorter hospital stays and may offer an economical and simplified management profile compared to standard anticoagulant treatment. Furthermore, rivaroxaban and dabigatran both show noninferiority to warfarin and cause fewer bleeding events at similar efficacies of preventing recurrent VTE. Indefinite anticoagulation can also be suggested for some provoked VTE patients, especially with persistent risk factors such as obesity, chronic immobilization, or genetic/inherited risk factors. The last group to consider for long-term anticoagulation and/or indefinite anticoagulation are those affected by a transient minor risk factor because of the propensity for thrombosis exacerbated by individual minor risks in this patient subgroup. Some of these minor risk factors include family history of VTE, surgery, trauma, or a brief singular moment of reduced immobility. Ultimately, regardless of the anticoagulant duration, the risk for bleeding should always be compared against the risk of recurrent VTEs. Moving forward, a list of validated and standardized risk factors for both repeated thrombosis and bleeding would be helpful to improve clinical decision making. Better patient subgroup classification and stratification along with clinical tools for identifying risks of recurrent VTEs and bleeding events will help clarify this controversy in the future
